# Ion-Pair Formation in Neutral Potassium-Neutral Pyrimidine Collisions: Electron Transfer Experiments

**DOI:** 10.3389/fchem.2019.00264

**Published:** 2019-04-18

**Authors:** Mónica Mendes, Beatriz Pamplona, Sarvesh Kumar, Filipe Ferreira da Silva, Antonio Aguilar, Gustavo García, Marie-Christine Bacchus-Montabonel, Paulo Limao-Vieira

**Affiliations:** ^1^Atomic and Molecular Collisions Laboratory, Centre of Physics and Technological Research (CEFITEC), Department of Physics, Universidade NOVA de Lisboa, Costa de Caparica, Portugal; ^2^Instituto de Física Fundamental, Consejo Superior de Investigaciones Científicas (CSIC), Madrid, Spain; ^3^Departament de Ciència de Materials i Química Física, Universitat de Barcelona, Barcelona, Spain; ^4^CNRS, Institut Lumière Matière, University of Lyon, Université Claude Bernard Lyon 1, Villeurbanne, France

**Keywords:** pyrimidine, negative ions, energy loss, time-of-flight, calculations

## Abstract

We report novel data on ion-pair formation in hyperthermal (30–800 eV) neutral potassium collisions with neutral pyrimidine (Pyr, C_4_H_4_N_2_) molecules. In this collision regime, negative ions formed by electron transfer from the alkali atom to the target molecule were time-of-flight mass analyzed and the fragmentation patterns and branching ratios have been obtained. The most abundant product anions have been assigned to CN^−^ and C_2_H^−^ and the electron transfer mechanisms are comprehensively discussed. Particular importance is also given to the efficient loss of integrity of the pyrimidine ring in the presence of an extra electron, which is in contrast to dissociative electron attachment experiments yielding the dehydrogenated parent anion. Theoretical calculations were performed for pyrimidine in the presence of a potassium atom and provided a strong basis for the assignment of the lowest unoccupied molecular orbitals accessed in the collision process. In order to further our knowledge about the collision dynamics, potassium cation (K^+^) energy loss spectrum has been obtained and within this context, we also discuss the role of the accessible electronic states. A vertical electron affinity of (−5.69 ± 0.20) eV was obtained and may be assigned to a $\pi_{3}^{{}^{*}}$(*b*_1_) state that leads to CN^−^ formation.

## Introduction

Radiation induced damage by low-energy electrons (<15 eV) has proven to be an efficient mechanism to promote local chemical changes when attaching to DNA/RNA molecular constituents (Boudaïffa et al., 2000). In such electron attachment process as a function of the phase and stage of aggregation, formed transient negative ion (TNI) can subsequently dissociate into stable fragment anions and neutral radical species (Sanche, 2009), where the latter may also trigger complex chemical damage within the biological environment. Another interesting aspect of such electron induced bond breaking pertains to the role of electron transfer processes which may be more prevalent under physiological conditions rather than free electron attachment processes (Wang et al., 2009). As far as electron induced processes are concerned, pyrimidine (Pyr) has been extensively studied as a prototype molecule of DNA and RNA building blocks (thymine, cytosine, and uracil) both in the gas (Baccarelli et al., 2011) and condensed phases (Sanche, 2005; Zheng and Sanche, 2018). Electron interactions with pyrimidine nucleobases are well-represented in the literature, where we note relevant experimental studies on electron transmission spectroscopy (Aflatooni et al., 1998), dissociative electron attachment (DEA) experiments (Huels et al., 1998; Ferreira da Silva et al., 2013), and electron impact ionization studies (Linert et al., 2012). More recently, site-selective bond excision of selected pyrimidines yielding the dehydrogenated parent anion upon electron transfer in collisions with neutral potassium atoms (Almeida et al., 2013a) and with low-energy electrons (Ptasinska et al., 2005) have been reported. N-site de-methylation in pyrimidine bases as studied by low-energy electron attachment and ab initio calculations gave a comprehensive description into the dynamics of the decaying transient anion and more precisely into the competition between dissociation and auto-detachment (Almeida et al., 2013b). Potassium-uracil/thymine ring cleavage enhancement was reported in electron transfer experiments and theoretical calculations (Almeida et al., 2014a). Studies on threshold behavior in metastable dissociation of multi-photon ionized thymine and uracil (Pandey et al., 2017) have been also investigated.

The topic of this contribution deals with electron transfer experiments with Pyr (C_4_H_4_N_2_) and within this context, a literature survey reveals that Nenner and Schulz experimental electron transmission spectroscopy data report the three resonances at 0.25 ($\tilde{X}$ ^2^*A*_2_), 0.77 (Ã ^2^*B*_1_), and 4.24 ($\tilde{B}$ ^2^*B*_1_) eV (Nenner and Schulz, 1975) while Modelli and Burrow (1983), and more recently Modelli et al. (2011), placed the three lowest electron affinities of π^*^ character at −0.39 [$\pi_{1}^{{}^{*}}$(*a*_2_)], −0.82 [$\pi_{2}^{{}^{*}}$(*b*_1_)], and −4.26 [$\pi_{1}^{{}^{*}}$(*b*_1_)] eV and a core-excited resonance at −5.5 eV. An unprecedented investigation on the effect of solvation on electron attachment to pure and hydrated Pyr clusters, has shown that hydration quenches all fragmentation channels in the pyrimidine molecule (Neustetter et al., 2015). Regarding theoretical calculations, we note a detailed study of the effect of the third π^*^ resonance on the angular distributions for electron-pyrimidine scattering (Mašín and Gorfinkiel, 2016) and electron affinities and ionization potentials of DNA radical ions (Sevilla et al., 1995). Total electron-scattering cross sections have been thoroughly investigated in several occasions (Baek et al., 2013; Fuss et al., 2013) together with differential cross sections for low-energy electron-impact excitation (Maljković et al., 2009; Jones et al., 2012a,b). Theoretical elastic and electronic excitation cross-sections and experimental electronic excitation cross-sections for electron collisions with pyrimidine have been reported by Mašín et al. (2012). Additionally, fragmentation of pyrimidine induced by core ionization by photoelectron-photoion-photoion coincidence (PEPIPICO) spectroscopy (Itälä et al., 2018) and absolute total and partial dissociative cross sections of pyrimidine by electron and proton intermediate impact velocities (Wolff et al., 2014), have been probed. The electronic state spectroscopy of pyrimidine has been comprehensively investigated by different methods, with threshold-electron excitation reported up to 12.5 eV (Pisanias et al., 1973), absolute cross-sections for electronic excitation have been obtained by electron impact up to 18 eV (Regeta et al., 2016) and absolute cross-sections by high-resolution VUV photoabsorption up to 11 eV (Ferreira da Silva et al., 2010). Low-energy (2–12 eV) electron vibrational and electronic electron-energy-loss (Levesque et al., 2005) and electron stimulated desorption from condensed pyrimidine (Ellis-Gibbings et al., 2017; Zheng and Sanche, 2018) have also been reported. Finally, a comparative study on the role of pyrimidine and water as underlying molecular constituents for describing radiation damage in living tissue, in terms of energy deposition (absorbed dose and stopping power) but also in terms of the number of induced molecular processes (Fuss et al., 2015) has been reported. Thus we consider that the present data on collisional electron-transfer induced dissociation of pyrimidine may serve for future applications in nanoscale models of radiation damage in DNA/RNA as we have recently proposed for the purines (Cunha et al., 2018a,b) and other biological relevant molecules as uridine (Almeida et al., 2014b) and small amino acids (Ferreira da Silva et al., 2016) just to mention a few.

Electron transfer experiments in atom-molecule collisions yielding ion-pair formation (reaction 1), an electron donor (K≡potassium) with low ionization energy (4.34 eV) delivers to the target molecule (Pyr) the extra charge, leaving it in a metastable state (Pyr^−#^):

(1) $$\begin{array}{l} \text{K}+\text{Pyr}\to {\left({\text{K}}^{+}{\text{Pyr}}^{-}\right)}^{\#}\to {\text{K}}^{+}+{\left(\text{Pyr}\right)}^{•}{}^{-\#}\to {\text{K}}^{+}\\ \;+{\left(\text{Pyr}-\text{X}\right)}^{-}\text{+X} \end{array}$$

which then dissociates into a stable fragment anion and a neutral (X) species. This process is dictated by non-adiabatic transitions between the neutral (K Pyr) and ionic (K^+^ Pyr^−#^) potential energy curves (and/or surfaces) involved in the collision (Kleyn et al., 1982), where the relative kinetic energy of the collision partners is greater than ΔE, the potassium atom ionization energy (IE) minus the electron affinity (EA) of the target molecule. For pyrimidine, a ΔE value <5 eV is obtained, meaning that the TNI can be formed with an excess of internal energy. Assessing the internal energy of the TNI requires detailed information of the angular distributions of products anions formed, viz. the products' velocity distribution. Pyrimidine has an electron affinity close to 0 eV (Nenner and Schulz, 1975) meaning that formation of a stable TNI may not be attainable within the μs detection window of the time-of-flight mass spectrometer. In the present experiments the lowest collision energy is 30 eV, the efficiency of such electron transfer process allows to explore complex reactions associated with concerted cleavage of several bonds.

In this manuscript we therefore report for the first time negative ion formation in neutral potassium-neutral pyrimidine collisions, together with K^+^ energy loss data and novel ab initio calculations to support the experimental findings. In the next sections, we describe our experimental methods and theoretical methodology. Afterwards, our results are presented and discussed and conclusions from this work are finally summarized.

## Experimental Methods

The crossed molecular beam setup used to study collisions of neutral potassium (K) atoms with neutral pyrimidine (Pyr), has been described in detail elsewhere (Ferreira da Silva et al., 2011; Almeida et al., 2013a), with recent modifications on the detection systems. Briefly, an effusive target molecular beam crosses a primary beam of fast neutral K atoms and the product anions are analyzed using a reflectron time-of-flight (TOF) mass spectrometer (KORE R-500-6). The K beam is produced in a resonant charge exchange chamber from the interaction of K^+^ ions from a potassium ion source (30–800 eV in the lab frame) with gas-phase neutral potassium atoms from an oven source. Residual ions were removed from the primary beam by electrostatic deflecting plates outside the oven. The neutral potassium beam's intensity was monitored using a Langmuir–Taylor ionization detector before and after the collection of each TOF mass spectrum and the beam energy resolution in the collision energy range was ~0.5 eV (FWHM) as measured with a hemispherical electrostatic energy loss analyser which characterized the K^+^ ion signal at a fixed energy, following K collisions with nitromethane (CH_3_NO_2_). The effusive beam of pyrimidine from an oven source was admitted into vacuum through a 1 mm diameter capillary where it was crossed with the neutral fast potassium beam. Negative ions formed in the collision region were extracted by a ~380 V cm^−1^ pulsed electrostatic field. The typical base pressure in the collision chamber was 6 × 10^−5^ Pa and the working pressure was 4 × 10^−4^ Pa. Mass spectra (resolution m/Δm ≈ 800) were obtained by subtracting background measurements (without the sample) from the sample measurements. Mass calibration was carried out on the basis of the well-known anionic species formed after potassium collisions with nitromethane (Antunes et al., 2010). Pyrimidine (Pyr) was supplied by Sigma Aldrich with a stated purity of ≥98%. Repeated freeze-pump-thaw cycles were performed before each spectrum collection. The extraction region and the TOF system were heated during the measurements in order to prevent any sample condensation and thus charge accumulation on the electrodes.

The entrance slit of the hemispherical energy analyser used in the K^+^ energy loss measurements is aligned in the forward direction with the neutral K beam's optical path. The analyser was operated in constant transmission mode, hence keeping the resolution constant throughout the entire scan where the energy resolution for the present measurements was ~0.6 eV in the lab frame. The energy loss scale was calibrated using the experimental threshold energy of formation (4.5 eV) from CN^−^, given that this is the most intense fragment anion formed in K-Pyr collisions.

## Theoretical Method

**Figure 1:**
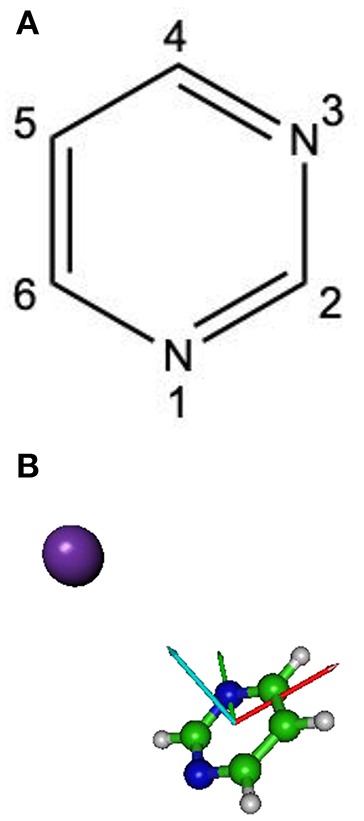
**(A)** Molecular structure of pyrimidine (Pyr); **(B)** orientation of the Pyrimidine + K collisional system; x, red; y, green, z, cyan.

**Figure 2:**
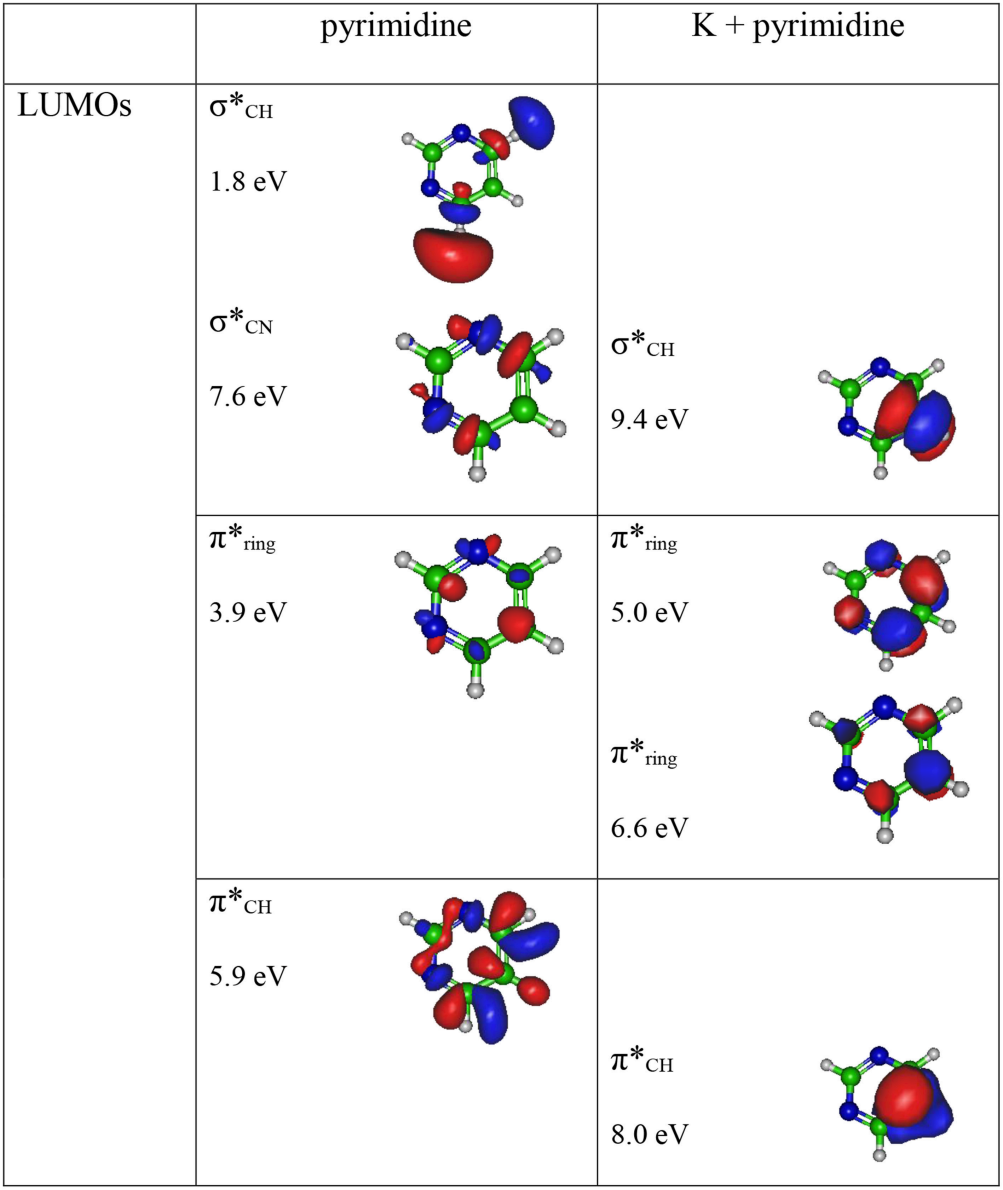
Calculated lowest unoccupied molecular orbitals (LUMOs) for pyrimidine (Pyr) and pyrimidine (Pyr) in the presence of a potassium perpendicularly to the pyrimidine ring (**Figure 1**). Energies in eV.

The theoretical description of the charge transfer process in the interaction of a neutral potassium atom with a selected nucleobase, is based on the evolution of the quasi-molecular system formed by the potassium projectile and the molecular target along the reaction coordinate within the framework of the molecular representation. We have implemented with success the one-dimension coordinate approximation in previous ion/neutral-biomolecule collision systems (Bene et al., 2008; Bacchus-Montabonel and Tergiman, 2012; Almeida et al., 2014a), where the atom-nucleobase collision system is thus treated as a pseudo-diatomic molecule evolving along the coordinate associated with the distance between the impinging atom and the nucleobase (Salem, 1982; Bacchus-Montabonel and Tergiman, 2011b). Within the frame of such approximation, we do not consider the internal degrees of freedom of the biomolecular target, which is reasonable since in very fast collision processes where nuclear vibrational and rotational motions are much slower than the collision time, these can be considered frozen during the collision. The geometry of pyrimidine has been optimized at the MP2 level of theory from the work of Bacchus-Montabonel and Calvo (2015). A perpendicular approach of the potassium atom, pointing at the center of the pyrimidine ring (see Figure 1) has been considered, as the charge transfer process has been clearly shown to be favored in such orientation for the case of pyrimidine targets (Bacchus-Montabonel and Tergiman, 2006, 2011a). The potential energy curves along the z reaction coordinate corresponding to the approach of the potassium atom perpendicularly to the pyrimidine ring have been calculated by means of ab-initio methods with the MOLPRO code (Werner et al., 2015). The pyrimidine target is kept frozen in its ground state geometry during the collision process. The calculation has been performed in Cartesian coordinates, with no symmetries. All electrons have been considered for C, N, and H atoms with the VTZ basis set, although the 18 core electrons of potassium have been treated through the ECP18sdf core-electron pseudopotential (Nicklass et al., 1995), with the corresponding basis set. The natural molecular orbitals for K–Pyr have been determined by CAS(3,11) state-averaged CASSCF calculations for the reaction coordinate *R* = 10 Å corresponding to the asymptotic region taking account the static electron correlation. The 1s orbitals of carbon, nitrogen and oxygen are treated as frozen cores. The resultant lowest unoccupied molecular orbitals (LUMOs) for pyrimidine are shown in Figure 2 together with the corresponding orbitals without the presence of potassium. The polarization by the potassium atom induces a global shift in energy of ~1.5–2.0 eV for the π^*^ orbitals and 2.0 eV for the σ^*^ orbital.

## Results and Discussion

**Figure 3:**
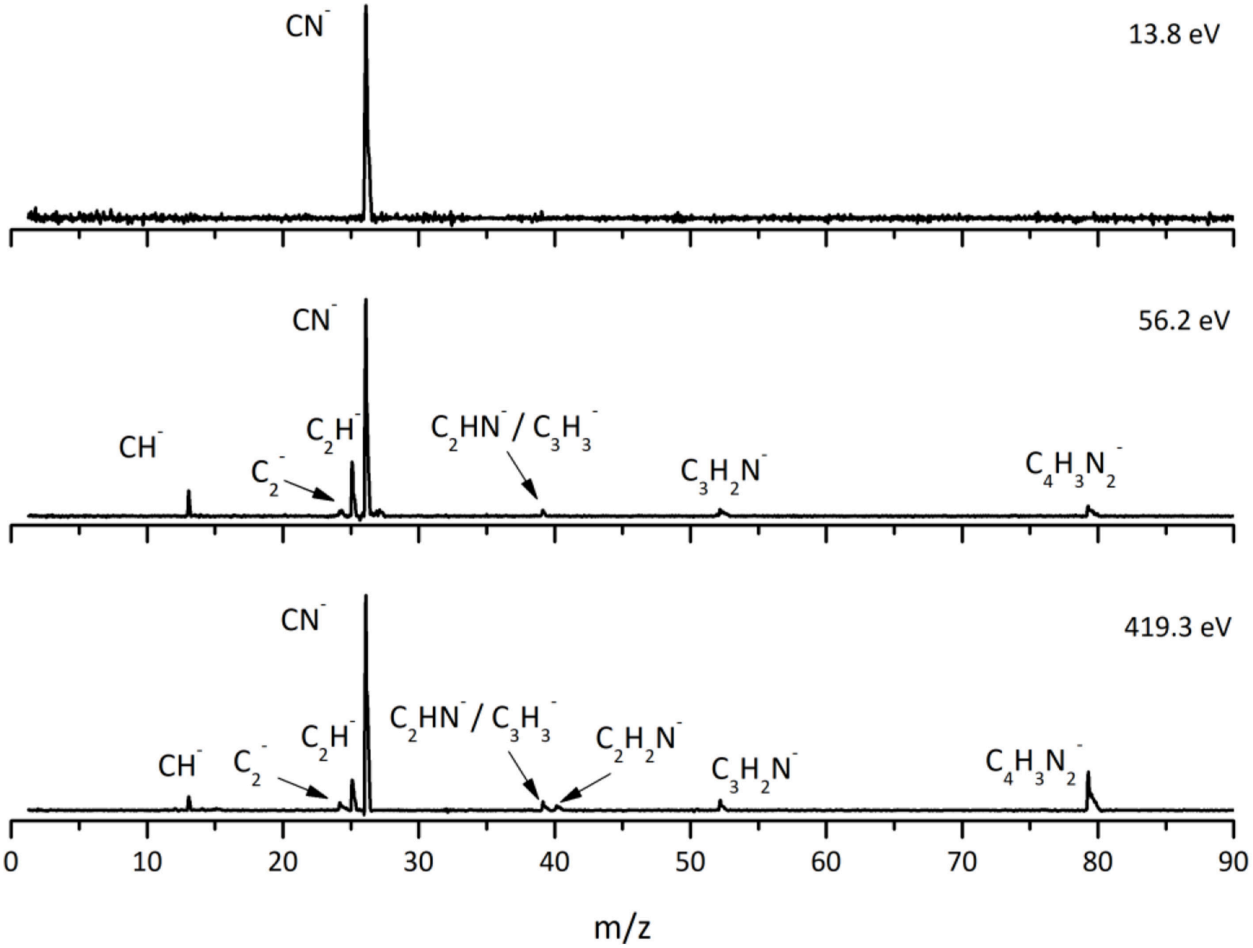
Time-of-flight negative ion mass spectra in potassium-pyrimidine (Pyr) collisions at 30, 100, and 700 eV lab frame energy (13.8, 56.2, and 419.3 eV available energy in the center-of-mass, respectively). See text for details.

**Figure 4:**
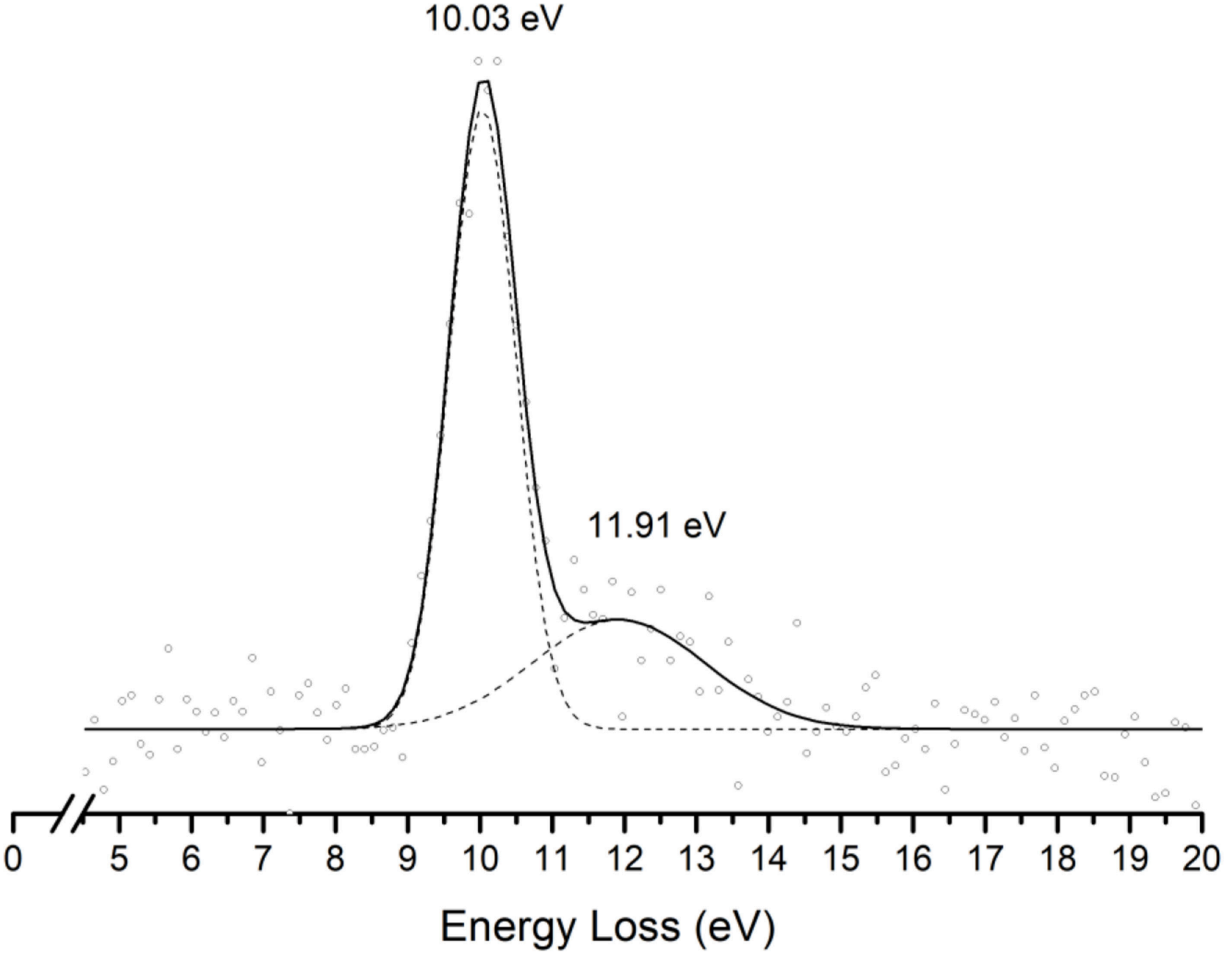
Energy loss spectrum of K^+^ ions measured in the forward direction in collisions of potassium atoms with pyrimidine (Pyr) at 111 eV lab frame energy (67.2 eV in the center-of-mass system). See text for details.

**Figure 5:**
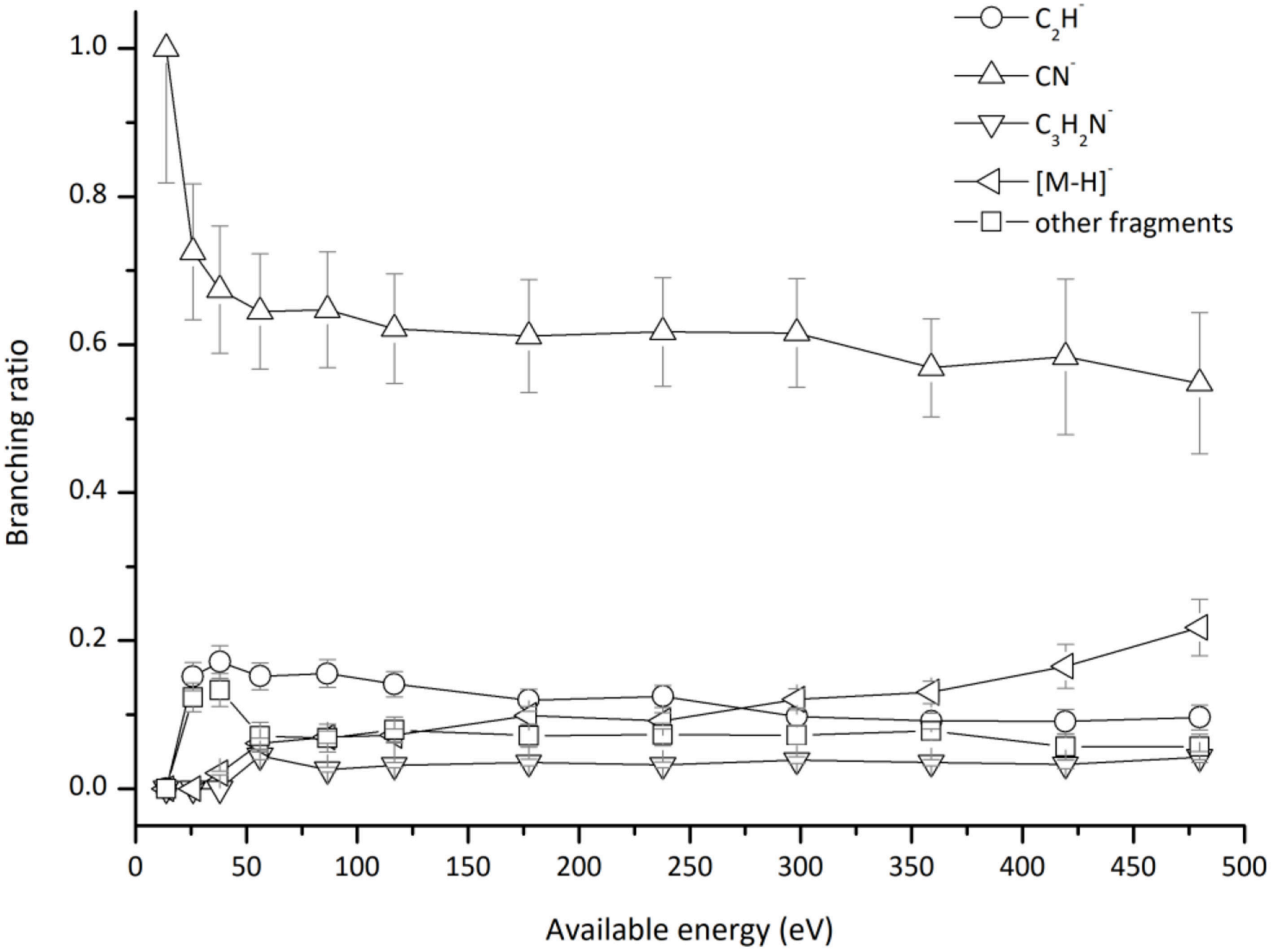
Pyrimidine (Pyr) branching ratios (fragment anion yield/total anion yield) of the main negative ions formed as a function of the collision energy in the center-of-mass frame. See text for details.

This is the first investigation on negative ion formation in electron transfer from neutral K atoms with Pyr combining experimental and theoretical methods to comprehensively analyse the full fragmentation pattern. Dissociative electron transfer TOF mass spectra were recorded at lab-frame collision energies of 30–800 eV (~14–480 eV in the center–of–mass frame and from now on referred as available energy). Table 1 is a compilation of all fragment anions detected and their proposed assignments in the wide energy range of collisions investigated. Figure 3 shows the Pyr negative ions TOF mass spectra recorded at 30, 100, and 700 eV lab frame energy (13.8, 56.2, and 419.3 eV available energy), while in Figure 4 we present the K^+^ energy loss spectrum measured in the forward direction in collisions of potassium atoms with pyrimidine at 111 eV lab frame energy (67.2 eV available energy). Branching ratios (BRs) for the major fragments of Pyr as a function of the collision energy are presented in Figure 5.

**Table 1:** Negative ions formed in potassium collisions with pyrimidine (Pyr).

**Mass (u)**	**Proposed assignment**
12	C^−^
13	CH^−^
14	${\text{CH}}_{2}^{-}$
15	${\text{CH}}_{3}^{-}$
24	${\text{C}}_{2}^{-}$
25	C_2_H^−^
26	CN^−^/C_2_${\text{H}}_{2}^{-}$
39	C_2_HN^−^/C_3_H${\text{H}}_{3}^{-}$
40	C_2_H_2_N^−^
50	C_3_N^−^
52	C_3_H_2_N^−^/C_2_${\text{N}}_{2}^{-}$
79	C_4_H_3_${\text{N}}_{2}^{-}$

A careful inspection of the TOF mass spectra reveals that they are dominated by the cyanide anion (CN^−^) and show no evidence of parent anion formation (Pyr^−^), which is expected since the vertical electron affinity of pyrimidine is −0.39 eV (Modelli et al., 2011). Another interesting aspect of the collision induced fragmentation pertains to the loss of different HCN units from the dehydrogenated parent anion of Pyr, (Pyr–H)^−^, yielding C_3_H_2_N^−^ (and/or the isobaric species C_2_N${}_{2}^{-}$), and C_2_H^−^ (Table 1), with such mechanism also reported in the case of potassium-adenine electron transfer experiments (Cunha et al., 2018a,b). The presence of the K^+^ ion in the vicinity of the TNI formed upon K + Pyr → (K^+^Pyr^−^) plays a significant role in the decomposition mechanism yielding particular fragmentation channels, which are different from those observed in DEA experiments (Neustetter et al., 2015). Such strong coulomb interaction in the collision complex (K^+^Pyr^−^) may delay autodetachment allowing time enough, in particular in the low-collision regime, for the excess energy in the TNI to be redistributed through the different available degrees of freedom enhancing a favorable fragmentation channel. In Pyr the fragmentation predominantly results in CN^−^ formation given the high electron affinity of the CN radical (3.8620 ± 0.0050) eV (NIST Chemistry WebBook, 2018). The ab initio calculations in Figure 2 show that the lowest-lying π^*^ states are considerably shifted to higher energies in the presence of a potassium atom in comparison to respective calculated MOs without the presence of K. During the electron transfer process, an electronic transition accessing a π^*^ state does not lead to direct cleavage of a bond unless a repulsive σ^*^ state is crossed diabatically. In the present experiments the available energy is larger than the threshold for electron transfer (ΔE = *IE*(K)−*EA*(Pyr) = 4.34 + 0.39 = 4.73 eV, with *IE*(K) being the ionization energy of the potassium atom and *EA*(Pyr) the Pyr electron affinity), and if the lifetime of the metastable ion is long enough, intramolecular energy redistribution may occur competing with direct dissociation. Such is possible if the nuclear wavepacket survives long enough along the reaction coordinate to allow diabatic coupling between the two states, i.e., π^*^ and σ^*^. This is discussed in the next sections within the scope of the different π^*^ and σ^*^ MOs involved in the formation of specific fragment anions.

### K^+^ Energy Loss Spectrum

The energy loss spectrum of K^+^ ions formed in the forward direction from the collision of potassium atoms with pyrimidine at 111 eV lab frame energy, is shown in Figure 4. Note that the lowest energy loss scale appears above ~ 4 eV to account for the potassium ionization energy, 4.34 eV. Two features are visible at 10.03 and 11.91 eV, the former more intense than the latter and with a full width at half-maximum (FWHM) of ~1.2 eV. The position of the maxima do not shift with the collision energy within ±0.2 eV. The main anionic yield from the TOF mass spectra at all collision energies is due to CN^−^ (Figures 3, 5). The energy loss ΔE at the maximum of the features is given by ΔE = *IE*(K) − *EA*_max_, which results on an electron affinity of (−5.69 ± 0.20) eV and (−7.57 ± 0.20) eV, assigned to $\pi_{3}^{{}^{*}}$(*b*_1_) and a $\pi_{CH}^{{}^{*}}$ core-excited resonance, respectively. This is in good agreement with the theoretical calculations presented in Figure 2.

### (Pyr–H)^−^

The dehydrogenated closed shell anion (Pyr–H)^−^ is formed through the ion-pair reaction:

(2) $$\begin{array}{l} \text{K}+\text{Pyr}\to ({\text{K}}^{+}{\text{Pyr}}^{-})\to {\text{K}}^{+}+{\left(\text{Pyr}\right)}^{\#-}\to {\text{K}}^{+}+\\ \;+{\left(\text{Pyr}-\text{H}\right)}^{-}\text{+H} \end{array}$$

which represents a direct cleavage of the (C–H) bonds and (Pyr)^#−^ stems for a TNI formed with an excess of internal energy. Formation of the parent anion with H abstraction has been reported in DEA experiments on pyrimidine through a core-excited resonance at 5.5 eV (Neustetter et al., 2015), where the three lowest π^*^ resonances do not contribute to such anion formation due to their short-lived character. Moreover, recent R-matrix calculations (Mašín et al., 2012) predict higher excited states which may contribute to (Pyr–H)^−^ formation. Pyrimidine BR as a function of the available energy (Figure 5), shows that (Pyr–H)^−^ yield only accounts for 10–20% of the total anions in the 50–480 eV energy region, and vanishes below the threshold of formation at ~26 eV. Such low anion yield in respect to the other fragment anions is in sharp contrast to the experimental observations in the low-energy collision regime of potassium atoms with DNA/RNA pyrimidines, thymine, and uracil (Almeida et al., 2013a; Ferreira da Silva et al., 2013). Such is certainly due to the different sort of molecular bonding where the presence of N–H bonds (in contrast to the C–H bond) lowers the threshold of the de-hydrogenated parent anion formation, which does not prevail in the case of pyrimidine.

In Figure 2 we show the three lowest calculated π^*^ MOs at 5.0 eV ($\pi_{ring}^{{}^{*}}$), 6.6 eV ($\pi_{ring}^{{}^{*}}$), and 8.0 eV ($\pi_{CH}^{{}^{*}}$) and at higher energy a σ^*^ resonance at 9.4 eV ($\sigma_{CH}^{{}^{*}}$) along the C5–H bond. Pyrimidine BRs in Figure 5 shows that (Pyr–H)^−^ cannot be produced <25 eV which can be related to an electron promotion to the $\pi_{ring}^{{}^{*}}$ orbitals yielding instead CN^−^. Accessing a $\pi_{CH}^{{}^{*}}$ state may be only possible by increasing the collision energy, and so (Pyr–H)^−^ formation certainly occurs through access of the $\sigma_{CH}^{{}^{*}}$ state via curve crossing. The present energy loss data provides evidence that the feature at 11.91 eV (see Figure 4), 7.57 eV for the electron affinity, is indicative of the vertical transition energy to the $\pi_{CH}^{{}^{*}}$ state, and the closeness with the $\sigma_{CH}^{{}^{*}}$ state allows us to specify the dominant pathway to dissociation. Alternatively, a direct initial transfer to the σ^*^ state and subsequent dissociation may be considered, playing a relevant role in the higher-energy collision region where the (Pyr–H)^−^ yield predominates in respect to the fragment anions produced through the $\pi_{ring}^{{}^{*}}$/$\pi_{CH}^{{}^{*}}$ resonances. Interesting to note the resonances that are prominent in the excitation functions for vibrational excitation, and peaking at ~10 eV, have been assigned to σ^*^ with pronounced C–H stretching character although ring breathing modes may be present (Regeta et al., 2016).

### ${\text{C}}_{3}{\text{H}}_{2}{\text{N}}^{-}/{\text{C}}_{2}{\text{N}}_{2}^{-},{\text{C}}_{2}{\text{H}}^{-}$

Hydrogen cyanide abstraction is operative from the de-hydrogenated parent anion leading to pyrimidine ring opening, with assignment of the fragment anions indicated in Table 1. Formation of C_3_H_2_N^−^ (and/or the isobaric ${\text{C}}_{2}{\text{N}}_{2}^{-}$) from potassium collisions with pyrimidine occurs at threshold >38 eV while for C_2_H^−^ above 14 eV in the center-of-mass frame (see Figure 5), the latter the second most intense fragment anion up to 250 eV. However, owing to C_2_H electron affinity, (2.969 ± 0.006) eV (Rienstra-Kiracofe et al., 2002) in contrast with −0.27 eV for C_3_H_2_N adiabatic electron affinity (from our present VTZ basis/CASSCF calculation), the former anion may prevail in the electron transfer induced decomposition of the pyrimidine molecule. We also observe a strong competition between C_2_H^−^ and (Pyr–H)^−^ formation which is enhanced above 250 eV. The LUMOs of Pyr in Figure 2 show relevant π^*^ antibonding character with nodes along the C–N bonds. Such electron spin densities are indicative of favorable bond breaking in particular where curve crossing in the diabatically frame description may be relevant (i.e., $\pi_{CH}^{{}^{*}}$/$\sigma_{CH}^{{}^{*}}$). However, at low collision energies (≤ 26 eV), the de-hydrogenated parent anion channel is not operative but is C_2_H^−^ although with modest intensity. The dominant K^+^ energy loss features peaks at 10.03 eV (Figure 4), 5.69 eV for the electron affinity and lends support to the electron spin densities suggesting that the electron may be initially transferred to the $\pi_{ring}^{{}^{*}}$ states. This (Pyr–H)^−^ suppression can be rationalized in terms of a slow collision process (>46 fs) enhancing a coulombic stabilization of the TNI by the proximate K^+^ ion, increasing the probability of intramolecular electron transfer that may favor dissociation or may favor autodetachment (supressing dissociation), certainly explaining the low yields observed in this energy region. As far as authors are aware, no DEA experiments to pyrimidine have reported these fragment anions. Within the collision energy range studied for pyrimidine, i.e., for the available energy (14–480 eV), such loss of HCN units is operative since the estimated threshold of the decomposition reaction requires −0.89 eV given that Δ_f_*H*_*g*_° (C_4_H_4_N_2_) = 196.65 kJ/mol (2.04 eV) (Lavorato et al., 2001), Δ_f_*H*_*g*_° (C_3_H_2_N) = −242 kJ/mol (−2.51 eV) (Huang et al., 2000), EA (C_3_H_2_N) = −26.05 kJ/mol (−0.27eV, from our present VTZ basis/CASSCF calculation), Δ_f_*H*_*g*_° (HCN) = 135.14 kJ/mol (1.4 eV), and Δ_f_*H*_*g*_° (H) = 218 kJ/mol (2.26 eV) (NIST Chemistry WebBook, 2018).

### CN^−^

The TOF mass spectra in Figure 3 and the BRs in Figure 5 are dominated by the cyanide anion in all collision energy range investigated. In sharp contrast to uracil and thymine collisional electron transfer experiments where the unimolecular decomposition process proceeds through the dehydrogenated parent anion as a precursor in the formation of fragments that require bond cleavages in the ring, namely CN^−^ (Ferreira da Silva et al., 2013), that is not the case in pyrimidine. In order to aid our understanding of the underlying molecular mechanisms and the accessed states that are responsible for CN^−^ formation in K-Pyr collisions, Figure 2 shows the three low-lying calculated π^*^ orbitals at 5.0, 6.6, and 8.0 eV. At higher energy a σ^*^ resonances at 9.4 eV is present with antibonding character along the C5–H bond. We now turn again our attention to the energy loss data in Figure 4 where the features have been assigned to transitions to electronic states through $\pi_{ring}^{{}^{*}}$ ($\pi_{3}^{{}^{*}}$(*b*_1_)) and a $\pi_{CH}^{{}^{*}}$ core-excited resonances at 5.69 and 7.57 eV, respectively. In the collision energy range investigated, CN^−^ is the major fragment anion and is mainly formed through an electron promotion to the $\pi_{ring}^{{}^{*}}$ orbitals. Such finding lends support to the theoretical prediction of the $\pi_{ring}^{{}^{*}}$ orbitals at 5.0 and 6.6 eV. Accessing the different π^*^ orbitals is achieved by increasing the collision energy and efficient bond breaking should proceed through access of σ^*^ states. However, the present calculations for K-Pyr do not predict any $\sigma_{CN}^{{}^{*}}$ states close in energy to the $\pi_{ring}^{{}^{*}}$ orbitals since these were performed without the presence of the K^+^ ion post-collision. From Figure 2, and in the case of the MOs for pyrimidine (left column), the $\sigma_{CN}^{{}^{*}}$ state shows strong antibonding character between C6–N1 and C4–N3 bonds. Owing to the similarity in the calculated electron spin densities between Pyr and K+Pyr in Figure 2, and apart from the differences in energies, we can anticipate a similar character for the C–N bonds. Notwithstanding, the proposed mechanism as suggested before in the case of the pyrimidines thymine and uracil (Almeida et al., 2011, 2014a), accounts for the initial access to one of the π^*^ states and subsequent intramolecular electron transfer into one of the highly antibonding σ^*^ states enhancing an effective ring-breaking pathway. Such is achieved in electron transfer studies since the presence of the K^+^ ion in the vicinity of the TNI may suppress autodetachment long enough for the fragmentation pathway successful competition (Almeida et al., 2011, 2014a).

## Conclusions

The present work provides the first comprehensive investigation of the decomposition mechanisms of neutral Pyr in collisions with neutral potassium atoms yielding ion-pair formation. The major negative ions formed have been investigated as a function of the available energy in the center-of-mass frame, and assigned to the cyanide anion, the de-hydrogenated parent anion, and fragment anions related to the pyrimidine ring opening due to abstraction of HCN units from (Pyr–H)^−^. The theoretical calculations reveal detailed information about the electronic structure of K+Pyr and hence provide insight into the electronic states that are most likely participate in the major fragment anion channels. We have also shown that ion-pair formation in collisions of potassium atoms with pyrimidine molecules, yields two different electronic states of the metastable parent anion. These states have vertical electron affinities of (−5.69 ± 0.20) and (−7.57 ± 0.20) eV, assigned to $\pi_{3}^{{}^{*}}$(*b*_1_) and a $\pi_{CH}^{{}^{*}}$ states, the latter accessible through a core-excited resonance.

## Author Contributions

MM, BP, and SK have run the experimental set up and collected the TOF mass spectra, energy loss data, and branching ratios. FdS was in charge of the data assessment and preliminary interpretation. M-CB-M was in charge of the theoretical calculations. AA, GG, and PL-V have been in charge of analyzing the data and paper writing together with M-CB-M.

### Conflict of Interest Statement

The authors declare that the research was conducted in the absence of any commercial or financial relationships that could be construed as a potential conflict of interest.
